# Keratoconus: diagnosis and management

**Published:** 2024-10-02

**Authors:** Rashmi Deshmukh, Daniel Gore

**Affiliations:** 1 Consultant: LV Prasad Eye Institute, Hyderabad, India.; 2 Consultant Ophthalmic Surgeon: Director of Refractive Surgery, Moorfields Eye Hospital, UK; 3 Honorary Clinical Lecturer, University College London, UK.

**A new treatment for keratoconus – corneal cross-linking – can slow or halt progression; however, early and accurate diagnosis remains essential.**

A definitive diagnosis of keratoconus is made using corneal topography and/or tomography.^1^ In settings where sophisticated imaging systems are not available, a simple Placido disk (or keratoscope) can be used. This reveals the shape of the front surface of the cornea (the topography) as a series of concentric rings (mires). In keratoconus, an irregular shape of the mires mirrors the irregular corneal curvature. An area of increased steepening is indicated where the reflected mires are close to each other (Figure 1). Placido-based imaging systems can provide detailed topographic maps, allowing you to assess corneal irregularities.

**Figure 1:**
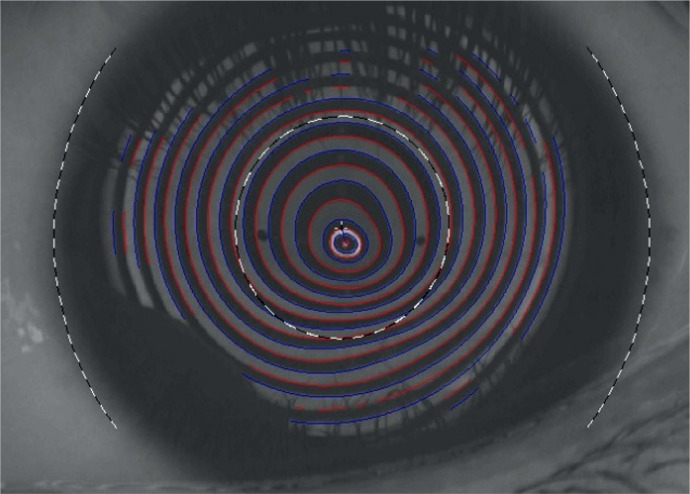
Image of Placido rings in keratoconus. The mires are closer to each other inferiorly, indicating an area where the cornea is steeper.

© ICEH CC BY-NC-SA 4.0

“In settings where sophisticated imaging systems are not available, a simple Placido disk (or keratoscope) can be used.”

Since these systems are based on reflection-based principles of topography, the posterior corneal surface is not directly mapped. However, it can be derived using mathematical calculations after carrying out ultrasound pachymetry to map corneal thickness. Two types of systems (available since the early 2000s) that can directly map the anterior and posterior surface of the cornea are Scheimpflug systems (e.g. Pentacam or Galilei) and scanning slit systems (e.g. Orbscan). These are generally more expensive.

Patients with keratoconus typically have asymmetric corneal steepening, coinciding with thinning and elevation in the same area (Figure 2).

**Figure 2:**
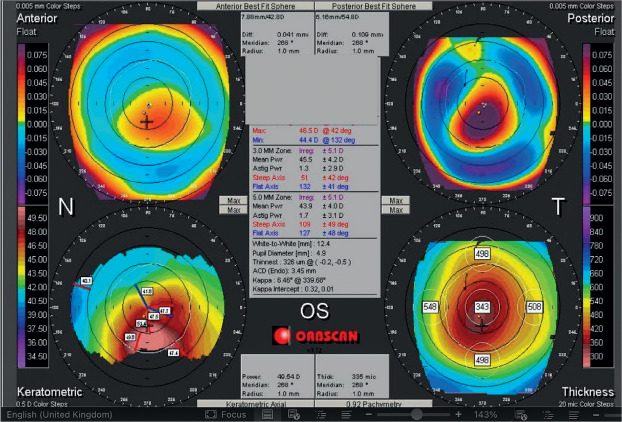
A quad map of a patient with keratoconus, produced using the Orbscan imaging system. Note the typical inferior steepening with abnormal elevation in the anterior and posterior elevation maps, as well as thinning in the same area.

© ICEH CC BY-NC-SA 4.0

In patients with advanced keratoconus, corneal optical coherence tomography (OCT) can show thinning. A relatively new device (MS-39, CSO Italy) combines Placido disk corneal topography with high resolution OCT-based anterior segment tomography. This can segment corneal thickness into epithelium and stroma (Figure 3).

**Figure 3:**
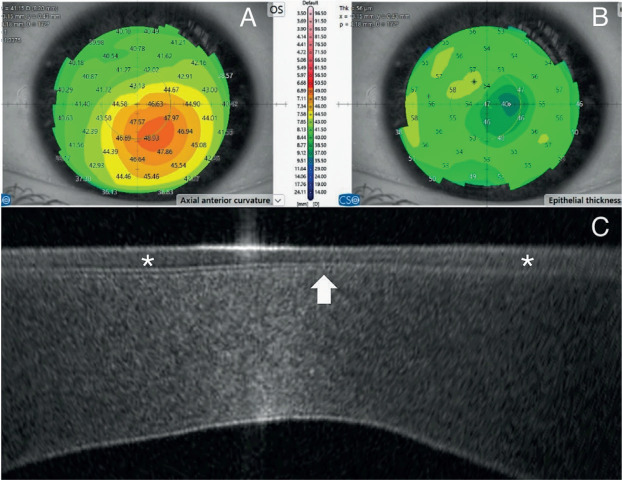
Combined Placido disk and OCT images of early keratoconus. **A.** Axial anterior curvature showing early ectasia. **B.** Epithelial thickness showing thinning over the corresponding area. **C.** Cross-sectional OCT image highlighting changes in epithelial thickness over the tip of the cone (white arrow) and at the base of the cone (asterisks).

© ICEH CC BY-NC-SA 4.0

Early keratoconus is often characterised by epithelial thinning over the tip of the cone, with corresponding thickening around the base of the cone as the epithelium tries to mask the irregular stroma. In these eyes, just looking at the front surface (topography), may miss early ectatic changes underneath. Cross-sectional corneal OCT is also useful to assess the depth of the scar in patients with more advanced keratoconus with stromal scarring, who are due to undergo keratoplasty. It is also useful in patients with hydrops, to assess the tear in Descemet’s membrane.

## Treatment

Historically, options to improve vision in keratoconus were limited to spectacles or rigid (gas permeable) contact lenses, corneal ring segment implants (since the 1990s), and corneal transplantation (penetrating or deep anterior lamellar keratoplasty) in patients with advanced keratoconus.

Wollensak first described corneal cross-linking (CXL) in 2003 as an approach to halt progression in cases of keratoconus and prevent worsening. Corneal cross-linking involves shining ultraviolet (UV) light at the cornea after the epithelium has been removed and riboflavin drops have been applied. This induces biochemical changes in the corneal stromal collagen, increasing the biomechanical stiffness, and halting further thinning and ectasia progression. Patients are typically monitored every 6–12 months with serial corneal scans to detect disease progression.

The standard approach is to offer corneal cross-linking to patients who are progressing. Corneal changes should be greater than the limits of repeatability for your scanning device (i.e., above machine noise). To improve the interpretation of scans, it is recommended to take three consecutive scans per eye per visit and ensure that contact lenses are removed for a consistent period prior to scanning (e.g., 1 week for soft contact lenses, 2 weeks for rigid gas permeable contact lenses). Unfortunately, there is no consensus on what constitutes a significant change, but an increase in keratometry values of more than one dioptre, or a reduction in corneal thickness of greater than 15 microns, might be considered significant.

Some patients are at a higher risk of progression, with young age considered the most important variable. In such high-risk patients, clinicians may opt to monitor more frequently (e.g. every 3 months) or consider offering corneal cross-linking at the point of diagnosis (i.e. without documented progression). The cornea naturally stiffens with age, and progression is unusual after people reach their mid-30s.

In patients with atopy, it is important to give them a short course of pre-operative steroid drops to make sure the ocular surface is quiet before corneal cross-linking is performed. Safety during pregnancy has not been established, but the hormonal changes during pregnancy can sometimes be associated with disease progression. Clinicians are advised to discuss these unknown risks with the patient, weighing them up against the potential benefits of preventing further deterioration in vision.

### Protocols

The initial protocol described, known as the Dresden protocol, involves epithelial debridement in the central 8–9 mm of the cornea and soaking it with riboflavin drops every 2 minutes for 30 minutes, followed by ultraviolet-A (UVA) 370 nm radiation exposure at 3 mW/cm^2^ for 30 minutes to achieve a surface dose of 5.4 J/cm^2^.

There have been several protocols since then, including accelerated and pulsed protocols.

The protocol of administering 10 minutes of ultraviolet light, with an irradiance of 9mW/cm^2^, is widely accepted and is used, for example, at LV Prasad Eye Institute in India.^2^ The protocol used at Moorfields Eye Hospital in London, UK is 30 mW/cm^2^ for 4 minutes, with a 1.5-second on/off cycle (total energy 7.2 J/cm^2^).^3^

Examples of corneal cross-linking devices include standard floor-mounted devices such as KXL (Glaukos Corp) and PXL (Peschke GmbH), as well as devices mounted on a slit lamp, such as the C-EYE device (EMAGine AG).

### Choice of riboflavin

The choice of riboflavin depends on the corneal thickness. All the above protocols are described for corneas with a minimum stromal thickness of 400 microns (which would be approximately 450 microns on tomography when the epithelium has not been removed). Isotonic riboflavin, made with dextran, is used in these patients.

### Stromal thickness and risk of endothelial damage

In patients with advanced keratoconus, where the stromal thickness is less than 400 microns, there is a potential risk of damage to the corneal endothelium. A number of approaches can be used in this situation, including using hypotonic riboflavin with physiological balanced salt solution, instead of dextran, which helps to increase the stromal thickness.^2^ Before starting the UV irradiation, use an intraoperative ultrasound pachymeter, if available, to confirm the stromal thickness to be more than 400 microns. Other eye units reduce the amount of energy being used for thinner corneas.

The risk of endothelial damage from corneal cross-linking appears to be low, and in practice many units would accept a corneal thickness of 400 microns or over on tomography (i.e. with the epithelium on, so that the thickness will be 350-370 microns with the epithelium off) for their normal cross-linking protocol. Units setting up their own cross-linking service are advised to do their own research and investigation to help decide what protocol to adopt.

### Postoperative care and pain management

Patients will experience pain in the initial 24-48 hours following the procedure. The pain can be severe, especially in younger in patients. Oral pain relief should be given, and some units give patients topical anaesthetic drops (e.g. 3 single-dose units) to use within the first 48 hours, although there is a risk of delayed healing if the eye is anaesthetised excessively. Although topical non-steroidal anti-inflammatory drugs (NSAIDs) have been described for pain relief, their use is not generally advised as there is risk of sterile infiltrates and stromal melts. Antibiotic drops or ointment are given for the first few days to prevent infection.

Postoperative monitoring is advised within the first week to monitor for complications. Early postoperative complications include poor epithelial healing, infective or sterile infiltrates, and central toxic keratopathy. Patients with a poor ocular surface from atopic disease are at particular risk of delayed epithelial healing. Delayed complications include haze and scarring. Spectacle prescription or contact lens trial may be given after 6 weeks of the procedure and these are likely to be needed for visual rehabilitation.

In patients with symptoms of poor visual quality due to irregular astigmatism and adequate corneal thickness, photorefractive keratectomy (PRK) can be used as an add-on procedure to regularise the cornea and improve visual quality. Epithelial removal using phototherapeutic keratectomy (PTK) has also been described.

“Like many conditions, the focus has shifted from treating the disease to preventing and diagnosing it at an early stage.”

## Summary

It has become apparent that keratoconus is more prevalent than initially believed. Like many conditions, the focus has shifted from treating the disease to preventing and diagnosing it at an early stage. Corneal cross-linking has proven to be transformative in the management of progressive keratoconus, and it has led to a reduction in the need for corneal transplant surgery where it is being used.

**Step-by-step guide for performing corneal cross-linking**

A clean room is required for the procedure; it is not necessary to perform corneal cross-linking in an operating room with air filtration.

Corneal cross-linking can be performed by a single staff member – traditionally by ophthalmologists, but in a number of eye clinics this now undertaken by trained nurses.

Corneal cross-linking devices are generally floor mounted, such as the KXL (Glaukos Corp.) and PXL (Peschke GmbH) or – less commonly – mounted on a slit lamp, such as the C-EYE device (EMAGine AG).

1. Select the preferred protocol. The **accelerated protocol** of 9mW/cm^2^ ultraviolet light for 10 minutes is widely used. (The total treatment time is 30 minutes: 5 minutes to set up and remove epithelium, 15 minutes for the riboflavin application, and 10 minutes of exposure to ultraviolet light.)
2. Instil topical anaesthetic drops into the eye(s) to be treated.
3. Debride the central 8-9 mm of corneal epithelium using a hockey knife or other blunt instrument. This is usually done with the patient lying down on a couch where the rest of the procedure will be performed, but it can be done using the slit lamp microscope. Epithelial debridement can be aided with applying 20% ethyl alcohol for 30 seconds in a corneal well, although this is not mandatory.
4. Soak the cornea with riboflavin for 10 to 20 minutes, applying drops every 1 to 2 minutes. In patients with thin corneas, where hypotonic riboflavin is being used, the stromal thickness can be checked according to the agreed protocol using a pachymeter.
5. Apply ultraviolet light of 370 nm using a cross-linking machine from an approved manufacturer. The light should be applied to the central part of the cornea, avoiding the limbus and potential damage to the limbal stem cells. Apply riboflavin drops every 2 to 5 minutes during irradiation.
6. At the end of the procedure, rinse the eye using sterile saline. A bandage contact lens (BCL) can be placed to improve comfort, but may increase the risk of sterile infiltrates forming. Alternatively, the eye may be padded along with antibiotic ointment.
